# Deep learning detects acute myeloid leukemia and predicts *NPM1* mutation status from bone marrow smears

**DOI:** 10.1038/s41375-021-01408-w

**Published:** 2021-09-08

**Authors:** Jan-Niklas Eckardt, Jan Moritz Middeke, Sebastian Riechert, Tim Schmittmann, Anas Shekh Sulaiman, Michael Kramer, Katja Sockel, Frank Kroschinsky, Ulrich Schuler, Johannes Schetelig, Christoph Röllig, Christian Thiede, Karsten Wendt, Martin Bornhäuser

**Affiliations:** 1 Department of Internal Medicine I, University Hospital Carl Gustav Carus, Dresden, Germany; 2 Institute of Circuits and Systems, Technical University Dresden, Dresden, Germany; 3 German Consortium for Translational Cancer Research DKFZ, Heidelberg, Germany; 4 National Center for Tumor Diseases (NCT), Dresden, Germany

**Keywords:** Acute myeloid leukaemia, Acute myeloid leukaemia, Translational research

## Abstract

The evaluation of bone marrow morphology by experienced hematopathologists is essential in the diagnosis of acute myeloid leukemia (AML); however, it suffers from a lack of standardization and inter-observer variability. Deep learning (DL) can process medical image data and provides data-driven class predictions. Here, we apply a multi-step DL approach to automatically segment cells from bone marrow images, distinguish between AML samples and healthy controls with an area under the receiver operating characteristic (AUROC) of 0.9699, and predict the mutation status of Nucleophosmin 1 (*NPM1*)—one of the most common mutations in AML—with an AUROC of 0.92 using only image data from bone marrow smears. Utilizing occlusion sensitivity maps, we observed so far unreported morphologic cell features such as a pattern of condensed chromatin and perinuclear lightening zones in myeloblasts of *NPM1*-mutated AML and prominent nucleoli in wild-type *NPM1* AML enabling the DL model to provide accurate class predictions.

## Introduction

A fundamental component in the diagnostic workflow of acute myeloid leukemia (AML) is cytomorphology [1]. The assessment of myeloblast counts and their morphology is essential for correct diagnosis, response assessment, and relapse detection. Cytomorphology may, in some cases, also lead to the suspicion of possible underlying genetics [2], e.g., in acute promyelocytic leukemia with t(15;17) and PML-RARα [3, 4] and AML with t(8;21), inv(16), or t(16;16) [5]. One of the most commonly mutated genes in AML is *Nucleophosmin 1* (*NPM1*). It plays a critical role in disease initiation and is utilized for molecular risk stratification in the recent European Leukemia Net 2017 (ELN2017) recommendations [1]. Mutated *NMP1* can be found in a third of all adult AML cases and up to 50–60% in AML with a normal karyotype [6, 7] and is considered a distinct disease entity in the current WHO classification [8]. So far, different morphological subtypes of AML according to the FAB classification [3] have been associated with different frequencies of *NPM1* mutations [9]. However, the interpretation of cytomorphologic image data is subjective, time-consuming, and suffers from intra- and inter-observer variability [10, 11]. Artificial neural nets (ANN) have demonstrated excellent capabilities in the processing of large quantities of image data [12]. Deep learning (DL) models are large-scale ANN consisting of a multitude of interconnected parallel processing units called artificial neurons [13, 14]. Especially convolutional neural nets (CNN) achieve outstanding results in image recognition [15]. These capabilities can be used for computer vision purposes in the diagnosis of acute leukemias [16–18].

In our study, we present a CNN-based scalable model that accurately distinguishes between AML cases and healthy subjects from digitalized bone marrow images. Further, our model accurately predicts *NPM1* mutation status from bone marrow cytomorphology and unveils distinct morphologic features for the prediction of *NPM1* mutation status.

## Methods

### Data set and molecular analysis

**Fig. 1:**
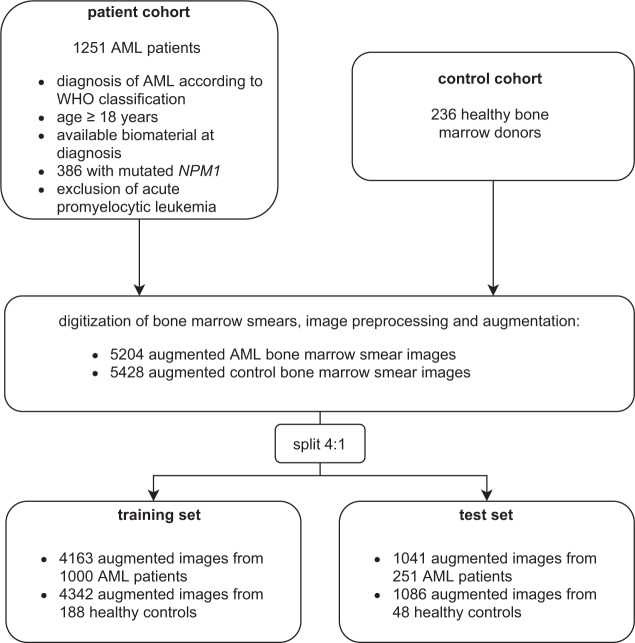
Study cohort. Patients were identified using retrospective chart review from previous multicenter clinical trials or the German Study Alliance Leukemia (SAL) registry. The control group was comprised of healthy bone marrow donors who underwent unrelated bone marrow donation at our center. Bone marrow smears (BMS) were digitized and sample size was increased by image augmentation (e.g. mirroring or rotating of images). Subsequently, samples were split into a training and test set with a ratio of 4:1.

We identified 1251 patients who have been newly diagnosed and treated with AML in the previously reported multicentric trials (AML96 [19]), AML2003 [20], AMLCG1999 [21], AML60+ [22], AMLCG2008 [23], and SORAML [24]) or from the multicentric patient registry of the German Study Alliance Leukemia (SAL, NCT03188874) via retrospective chart review. Eligibility criteria for the AML cohort were newly diagnosed AML according to WHO criteria [8], age ≥18 years, and available biomaterial at initial diagnosis. A control cohort was comprised of 236 bone marrow samples from healthy bone marrow donors who underwent bone marrow donation at our center. Figure 1 shows the set-up of the study cohort and the split of augmented image data between training and test set (4:1). All mentioned studies were previously approved by the Institutional Review Board of the Technical University Dresden. All participants gave their written informed consent according to the Declaration of Helsinki. The preparation of squash slides was performed from anticoagulated bone marrow by experienced laboratory technicians within 2 h after the sample was taken, as recommended by WHO guidelines [25]. Sample staining was performed with the May–Grunwald–Giemsa method [26]. Screening for *NPM1* mutations was performed as described previously [27] and a 5% variant allele frequency (VAF) mutation cut-off was used. High-resolution pictures of representative regions of the bone marrow smears (BMS) were taken using the Nikon Eclipse E600 microscope (50-fold magnification) with the Nikon DSFi2 mounted camera and Nikon Imaging Software Elements D4 for image processing. Corresponding regions of interest were manually selected by hematologists and measured 0.1775 × 0.1325 mm. For selection of archived BMS, image acquisition, and upload of images to the database, 10 min of manual labor were needed per case. Per case, a median of 168.5 cells were captured (interquartile range: 124–217). Samples were randomly assigned to either a training or a validation set with a split of 4:1.

### DL model

**Fig. 2:**
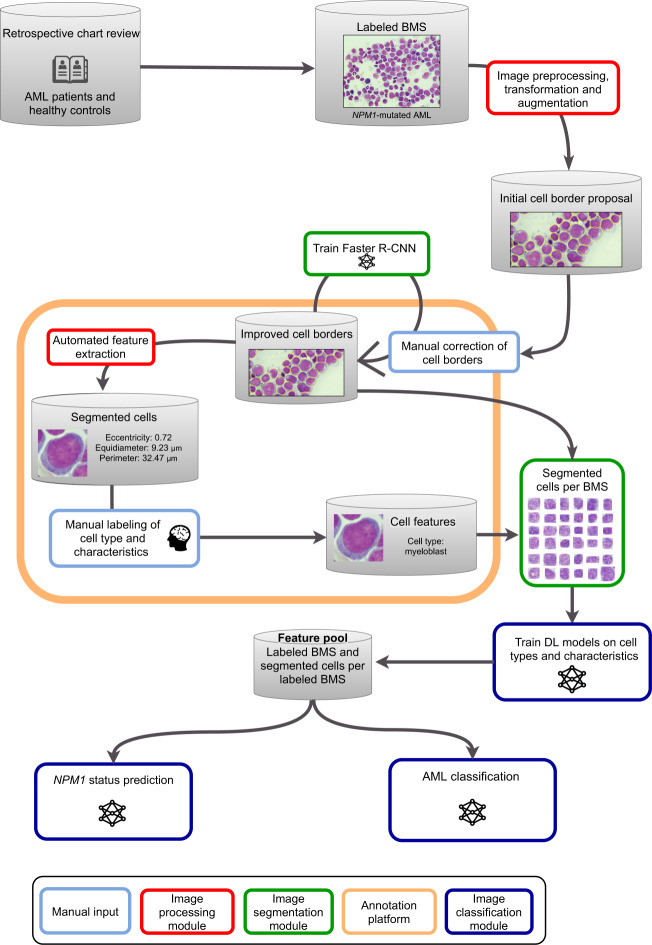
Schematic workflow of the multi-step deep learning platform. AML patients and healthy controls were identified via retrospective chart review. Representative pictures of bone marrow slides (BMS) were taken and labeled according to diagnosis and *NPM1* mutation status. Subsequent image preprocessing, transformation and augmentation led to initial cell border proposals by the Faster Recurrent Convolutional Neural Net (FRCNN) that were manually corrected in order to improve cell borders. The FRCNN was trained iteratively to improve cell border proposals. Automated feature extraction on segmented cells by the image processing module determined characteristics like cell perimeter or nucleus-to-cytoplasm ratio (N:C). Segmented cells were manually labeled according to cell type and characteristics. Manual cell segmentation and labeling was performed with a modified version of the VGG image annotator tool. Thereby, a feature pool of BMS, segmented and labeled cells was generated to iteratively train the image classification module with a split of 2:1 between training and validation set using threefold cross-validation.

A multi-step machine learning workflow with individual DL models for different tasks was set up as shown in Fig. 2. Step 1: BMS were digitalized and uploaded to an online segmentation and labeling platform that we developed for the purpose of this work. A human-in-the-loop cell segmentation approach was performed by hematologists with a Faster Region-based Convolutional Neural Net [28] (FRCNN). First, initial segmentation was done with the VGG Image Annotator [29] tool. Then, the FRCNN was trained with the segmented images and created new segmentation proposals for unsegmented images which were manually corrected by hematologists. The loop was closed by the refinement of segmentation proposals and repeated network training. A quarter of cases was segmented using this human-in-the-loop approach while for the remainder of cases the FRCNN worked autonomously without the need for human intervention for re-segmentation of cells. This way, segmentation quality improved substantially over iterations eliminating the need for manual segmentation in the following unsegmented images. Additionally, hyperparameter optimization was performed automatically using the Optuna [30] framework with a predefined hyperparameter space. Step 2: Feature extraction was performed manually by hematologists. In all, 8500 individual cells were labeled according to lineage, cell type, and characteristics like Auer rods. Features like cell size, eccentricity, and color range were automatically derived by the computer vision algorithms. Step 3: For distinction between AML and healthy control samples based on segmented images, we trained a multitude of DL models for binary predictions of cell types and characteristics that expressed results as ratios (e.g. ratio of myeloblasts among all cells or features such as presence or absence of Auer rods). The aggregated results given by these individual models were used as input for an Ensemble Neural Net (ENN) for final classification decisions. Model architecture for the distinction between AML and healthy control samples was based on the Xception CNN [31] utilizing transfer learning. Xception architecture was modified to receive BMS images (2560 × 1920 pixels) as input at the top level. Fully connected output layers for the 2048-dimensional output vectors of the core Xception architecture were established via hyperparameter optimization. Hyperparameters differed between individual models. Step 4: For *NPM1* status prediction, transfer learning with a ResNet50 [32] pretrained on ImageNet was utilized on BMS images. The ResNet50-architecture was modified to use larger input sizes (2000 × 1500 pixels) and the output layer was reshaped to a fully connected layer with two neurons to accommodate the binary classification problem before retraining. Hyperparameter optimization was performed for learning rate, learning rate gamma, momentum, and weight decay. Occlusion sensitivity maps were used to derive information from classification decisions for *NPM1* status prediction. DL models were implemented in Python version 3.7.9 with Keras version 2.3.0, TensorFlow version 2.1.2, and PyTorch version 1.5.0. Computations were performed using a high-performance computing system.

### Model performance and statistical analysis

For performance analysis of the classification models, we used precision–recall curves and receiver operating characteristics (ROC) with the area under the curve (AUC). Precision is the fraction of true positives among all positive predictions of the DL model while recall is the fraction of all positive predictions of the DL model among all relevant events. The final models were evaluated on the validation set that was withheld from model training. To compare *NPM1* VAF the Mann–Whitney *U* test was used. Computational and statistical analysis was performed in Python (version 3.7.9) and R (version 4.0.3).

## Results

### DL accurately distinguishes between BMS of AML patients and healthy bone marrow donor samples

**Fig. 3:**
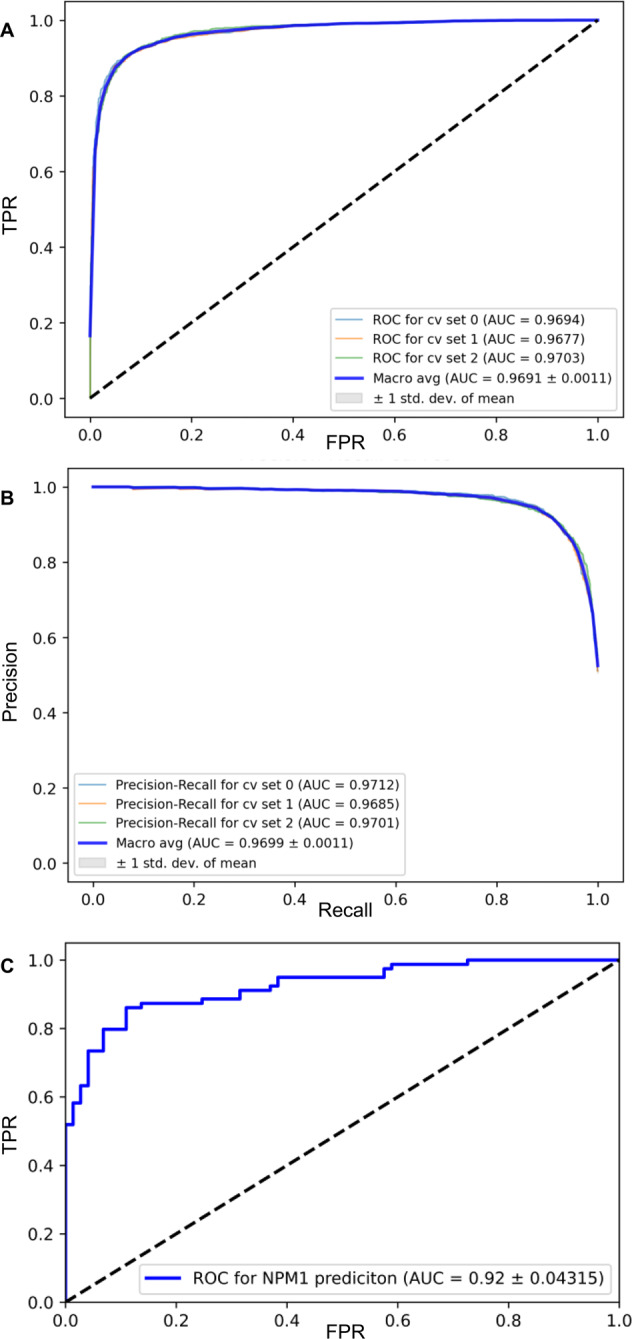
Performance measures of the AML classification model and *NPM1* prediction. **A** Precision–recall curve and **B** receiver operating characteristic (ROC) for the binary classification of AML vs. healthy bone marrow donor samples from bone marrow slide images obtained by threefold cross-validation (c.v.). Results of the individual c.v. sets are shown in the respective boxes and depicted as light blue, orange, and green graphs. Performance was measured calculating the area under the curve (AUC) for individual validation sets by threefold c.v. and averaging results (Macro avg, blue graph) with one standard deviation (SD) of the mean. **C** ROC for the binary classification of *NPM1* mutation status on bone marrow slide images with fivefold cross-validation. TPR true positive rate, FPR false-positive rate.

We retrospectively identified 1251 AML patients, 386 of which harbored mutated *NPM1* according to molecular analyses. Detailed information on patient characteristics and controls is provided in Table 1. A total of 94,162 individual cells were manually segmented to iteratively train the FRCNN. Subsequently, automatic cell segmentation with the FRCNN achieved a mean average precision and a mean average recall of both 0.97 at an intersection over union ratio of 0.5. Inaccuracies were mostly due to overlapping cells. We then applied a CNN-based binary classification model on the previously segmented images to distinguish between cell types and characteristics and aggregated results were used by an ENN to distinguish between AML and healthy donor samples. We found this multistage approach to substantially increase accuracy over simple whole image classification with only one CNN. Single cell-based disease status prediction was tested, but did not yield satisfactory results (AUROC 0.53). To adjust for the moderate sample size and to balance the data set, simple image augmentation techniques like linear transformations or adjustment of color channels and brightness range BMS images were applied. Thereby, we reached an augmented sample size of 5204 AML and 5428 non-AML (healthy donor) BMS images. To prevent overfitting, we used a pooling dropout of 0.25 as suggested by automated hyperparameter optimization. The AML classification model achieved an average AUC for the precision–recall curve of 0.9691 (95% CI: 0.9669–0.9713; Fig. 3A) and an average AUROC of 0.9699 (95% CI: 0.9677–0.9721; Fig. 3B) with a corresponding micro-average accuracy of 0.91. Table 2A shows the distribution of correctly and incorrectly identified AML and control samples in the validation set.

**Table 1:** Patient characteristics.

Parameter	All AML samples	NPM1-mutated AML	NPM1 wild-type AML	Bone marrow donors
*N*	1251	386	865	236
Age, median (IQR)	57 (38–67)	57 (49–66)	54 (38–64)	31 (25–39)
**Sex,** ***n*** **(%)**				
Male	668 (53.4)	173 (44.8)	495 (57.2)	70
Female	583 (46.6)	213 (55.2)	370 (42.8)	30
**AML type,** ***n*** **(%)**				
De novo	969 (77.7)	339 (88.3)	630 (73)	/
sAML	177 (14.2)	24 (6.2)	153 (17.7)	/
tAML	101 (8.1)	21 (5.5)	80 (9.3)	/
**ELN2017 risk,** ***n*** **(%)**				
Favorable	380 (33)	277 (78)	111 (13.8)	/
Intermediate	521 (45.3)	75 (21.1)	446 (55.5)	/
Adverse	249 (21.7)	3 (0.8)	246 (30.6)	/
PB blasts, median (IQR)	23.5 (4–60)	38 (11–72.5)	18 (2–51)	/
BM blasts, median (IQR)	60.5 (40–80)	73.5 (54–85)	54 (37–75)	/

Patient characteristics for the AML and control (bone marrow donors) cohort. The AML cohort is further subdivided by *NPM1* mutation status. AML type was defined according to the WHO 2016 classification. *sAML* secondary AML, *tAML* therapy-associated AML, *PB* peripheral blood, *BM* bone marrow, *N*/*n* number, *IQR* interquartile range.

**Table 2:** Classification accuracy in the validation set.

**(A) AML vs. control**		**Prediction by deep learning**	
		**Healthy control**	**AML**
Ground truth			
Healthy control		43 (89%)	5 (11%)
AML		28 (13%)	223 (87%)
**(B) mNPM1 vs. wtNPM1**		**Prediction by deep learning**	
		**wt*NPM1***	**m*NPM1***
Ground truth			
wt*NPM1*		149 (86%)	24 (14%)
m*NPM1*		11 (14%)	66 (86%)

The model was tested on a validation set of (A) 48 healthy controls and 251 AML patients. Misclassifications were 11% false positives (healthy controls misclassified as AML) and 13% false negatives (AML patients misclassified as healthy). For binary classification of *NPM1* mutation status, the validation set was comprised of 77 patients with mutated (m)*NPM1* as well as 173 patients with wild-type (wt)*NMP1*. Misclassifications were 14% false positives (wt*NPM1* patients predicted to have m*NPM1*) as well as 14% false negatives (m*NPM1* patients predicted to have wt*NPM1*). It is to be noted, that dysbalanced patient/control numbers were accounted for by image augmentation to balance the data sets for proper training of the classifiers.

### DL accurately predicts *NPM1* mutation status and unveils morphologic features

**Fig. 4:**
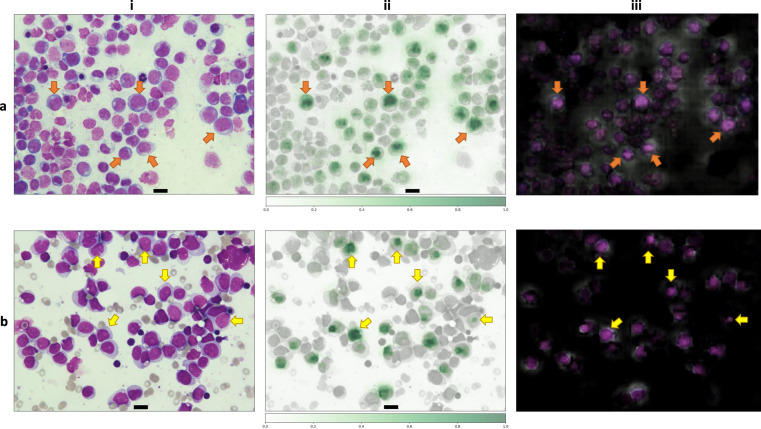
Application of occlusion sensitivity maps to detect features derived by deep learning for the prediction *NPM1* mutation status in AML. Representatives images from bone marrow samples from **a** a patient with mutated *NPM1* AML and **b** a patient with wild-type *NPM1* AML. (i) Bone marrow aspirates were stained with the May–Grunwald–Giemsa method. Pictures were taken at 50-fold magnification using a Nikon ECLIPSE E600 microscope with a mounted Nikon DSFi2 camera. Images were processed using Nikon Imaging Software Elements D4. Scale bar = 10 µm. (ii) Heatmap of occlusion sensitivity maps generated in Python. The scale indicates the importance of certain image areas for correct class prediction: the more intensively green an area is the more important it is for correct class prediction. The heatmap shows a cell-specific class evaluation by the deep learning model. (iii) Masked image overlay of (i) and (ii) generated in Python showing areas the deep learning model focused on. Bright image areas are more important for class predictions. The orange arrows in (**a**, i) point to examples of cells with condensed chromatin and perinuclear lightening zones the deep learning model picked up for prediction of mutated *NPM1*. The yellow arrows in (**b**, i) point to examples of cells with prominent nucleoli the deep learning model picked up for prediction of wild-type *NPM1*.

**Fig. 5:**
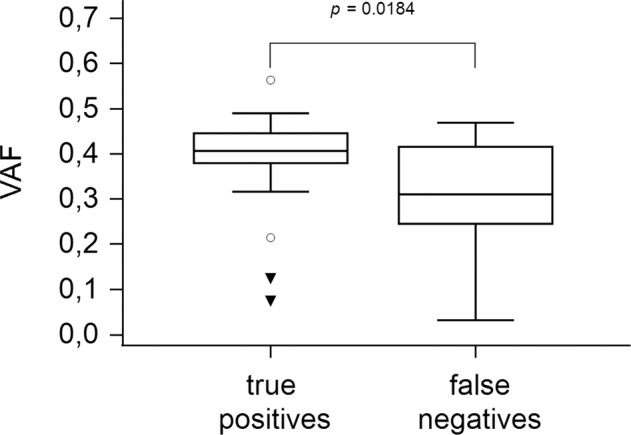
Variant allele frequency of *NPM1* true positives and *NPM1* false negatives. Box-and-whisker plot for variant allele frequency (VAF) of mutated *NPM1* AML samples that were correctly identified as such by the deep learning model (true positives) and those that were misclassified (false negatives) compared by the Mann–Whitney *U* test.

Further, we investigated whether DL could predict the mutational status of *NPM1* from bone marrow morphology. BMS image classification at a 50-fold magnification was performed using a ResNet50-architecture using transfer learning. Mirroring and random cropping plus resizing was used to increase sample size and to balance the data. Weight decay of 0.0003 is utilized to prevent overfitting and the data was divided into training and test set with a split of 4:1. The model achieved an accuracy of 0.86 for *NPM1* prediction and an AUROC of 0.92 (95% CI: 0.8768–0.9631; Fig. 3C). Table 2B shows the distribution of correctly and incorrectly identified NPM1-mutated and NPM1 wild-type samples in the validation set. Classification on single cells compared to whole image classification did not improve accuracy. To identify key morphological features that led the DL model to the prediction of the respective mutation status we used occlusion sensitivity maps (Fig. 4). This method iteratively blocks pixels of an image from being evaluated by the DL model for classification, which may reduce its predictive capabilities. Thereby, image areas that are essential for high accuracy can be detected as they greatly reduce model performance when being blocked (Fig. 4 ii, iii). By analyzing the heatmaps, we observed that the prediction of mutated *NPM1* was associated with a pattern of condensed chromatin and perinuclear lightening zones in myeloblasts (Fig. 4a, orange arrows indicate examples). The prediction of *NPM1* wild type was driven by prominent nucleoli (Fig. 4b, yellow arrows indicate examples) which could only very rarely be observed in samples with mutated *NPM1* and in that context led to misclassification (false negatives). We further analyzed patient samples from the validation set grouped by *NPM1* mutation status and true or false predictions given by the DL model regarding clinical and molecular data. *NPM1*-mutated AML samples that were correctly identified by the model (true positives) had a significantly higher median VAF than *NPM1*-mutated AML samples that were misclassified (false negatives) (true positives: 0.41 [95% CI: 0.39–0.42] vs. false negatives: 0.31 [95% CI: 0.1–0.42], *p* = 0.018, Fig. 5). Further, the rate of patients with therapy-associated AML (tAML) was significantly higher among false negatives compared to true positives (27.3% vs. true positives: 4.1%, *p* = 0.02) and false negatives had a significantly lower median white blood cell count (WBC) (false negatives 13.16 GPt/l [IQR:2.25–28.57] vs. true positives: 37.48 GPt/l [IQR:17.84–84.95], *p* = 0.007) and a trend for lower blast counts in peripheral blood (false negatives 25.5% [IQR: 4.75–38.5] vs. true positives: 52.5% [IQR: 16–75.25], *p* = 0.062). No significant differences for age, sex, ELN2017 risk category, absence or presence of a complex karyotype, bone marrow blast count, Hb, and platelet count were detected. For patients with wild-type *NPM1* AML, there was a trend for lower median WBC for true negatives (3.54 GPt/l [IQR: 1.52–19.82] vs. false positives 11.3 GPt/l [IQR:3.95–36.849], *p* = 0.095), but age, sex, ELN2017 risk category, AML type (de novo, secondary or therapy-associated), absence or presence of a complex karyotype, bone marrow or peripheral blast count, Hb and platelet count showed no differences. As another internal sanity check, we applied the pretrained classifier to the healthy bone marrow donor image data set and found that 214/236 (91%) of cases were correctly identified as NPM1 wild type while only 22/236 (9%) were labeled as NPM1 mutated (false positives). However, we want to point out that the NPM1 classifier has never been trained on healthy controls. Considering its AML-specific training, the very low false-positive rate on newly presented and differently structured image data of healthy controls underlines the distinct morphology picked up by the classifier for correct predictions in NPM1-mutated AML. Interestingly, when we reviewed patient chart data and molecular results in the validation set, we found one sample that was incorrectly labeled as mutated *NPM1*, but was in fact wild-type *NPM1*, and the corresponding BMS image was correctly identified as such by the DL model.

## Discussion

We here present a machine learning approach for cell segmentation and image classification which provides a fast, scalable, and highly accurate method to identify AML samples from bone marrow cytomorphology. Our FRCNN achieved a cell segmentation accuracy of 0.97 from BMS. The binary classification model showed an AUC of 0.97 for both the ROC and the precision–recall curve and a micro-average accuracy of 0.91 distinguishing between AML and healthy bone marrow donor samples. Our model can potentially be applied in initial diagnosis when a case of suspected AML is evaluated upon first contact in a hematologic center. It operates autonomously once BMS images are uploaded and detects AML with high accuracy. The model could operate synchronously with lab technicians to flag cases that are highly suspicious of AML for fast evaluation by experts while results from other diagnostic procedures like flow cytometry, cytogenetics, and molecular genetics are still pending. However, a human-in-the-loop approach is still needed as we manually selected representative regions of the BMS for evaluation by the DL model. Also, it is to be noted that bone marrow donors in our cohort were substantially younger than AML patients. Increased age is associated with observable changes in the bone marrow microenvironment such as cellularity, proliferative activity, and apoptosis [33] and such systematic differences could introduce bias to a CNN classifier which needs to be taken seriously not only in our use case but also considering other applications of more subtle changes in bone marrow morphology. Further evaluation of the model using more diverse multicenter data is warranted. Another limitation is the necessity for manual selection of BMS areas representative for disease classification by human judgment. Since this is a potential source of bias, future work will focus on implementing whole slide imaging and an automatization of region-of-interest selection given recent advances such as DL-based automated focusing [34]. Further automatization of BMS development can be achieved using automated BMS staining devices [35].

Furthermore, we used DL to predict the mutation status of *NPM1* from cytomorphology alone. For *NPM1* prediction our DL model achieved a high accuracy of 0.86 in predicting mutation status. AML with mutated *NPM1* has previously been associated with cup-like blast morphology [36, 37]. When analyzing the features that the model used for *NPM1* classification with occlusion sensitivity maps, we found so far unreported features like a pattern of condensed chromatin accompanied by perinuclear lightening zones for *NPM1*-mutated blasts. We observed prominent nucleoli in myeloblasts as a feature the DL model derived to predict wild-type *NPM1* AML while these could only rarely be observed in *NPM1*-mutated AML samples and then led to misclassification by the model. Further, we found a significantly higher VAF in *NPM1* true positives while the group of false negatives was comprised of a significantly higher rate of tAML. Wild-type NPM1 serves as a critical structural protein of the nucleolus, but mutations lead to a delocalization to the cytoplasm [38]. This process is partially triggered by insertions causing a frameshift of the C-terminal end of NPM1 and the formation of nuclear export signals [39, 40]. Weakened anchoring and predominant export signals subsequently result in increased nuclear export of NPM1 [38]. Arguably, a cytomorphologic correlate of this process may be the presence of prominent nucleoli in wild-type *NPM1* AML and the absence thereof in mutated *NPM1* AML—both detected as highly predictive features by our DL model.

Our study shows that DL can derive morphologic features from cytomorphology that predict mutation status. Future work will focus on other clinically important mutations and their morphologic imprint that DL may be able to pick up. In line with our findings, a recent study showed that DL can associate the morphology of myelodysplastic syndromes (MDS) with distinct genetic imprints [41]. However, in order to be integrated into clinical practice, machine-learning models need to be accurate and generalizable. As their development is complex, collaborations between physicians and software engineers is needed in an iterative approach to increase model performance. Since the majority of recently proposed machine-learning models—along with our model—are built on retrospective data, future studies will have to implement such models in a prospective setting to confirm their diagnostic value [42]. Due to the heterogeneity of cell morphology as well as close proximity of cells, disease classification from bone marrow is much more complex than in peripheral blood. Our use case to delineate AML from healthy bone marrow serves as groundwork for more complicated applications of CNNs in bone marrow morphology. AML is defined by bone marrow blast count [1] and CNNs can use a ratio of blasts to accurately detect AML. However, more complex use cases such as reactive bone marrow changes, benign disorders such as vitamin B_12_ deficiency, or hematologic neoplasms such as MDS are associated with subtle changes of cell morphology [43, 44]. In this scenario, CNNs have to be trained to accurately detect and assign such morphologic changes to the respective disorders. Mori et al. [45] recently used CNN-guided detection of decreased granules—one of the most common dysplastic changes in MDS—and report high accuracy for their classifier based on the ResNet-152. Accordingly, integrated analysis of more complex morphologies can potentially be achieved by feature engineering using a knowledge bank of expert-annotated cells with sufficiently sized training sets per morphologic feature (conceivably in the four- to five-digit number range). Since many hematologic neoplasms are rare disease entities, the development of such a large database requires extensive cooperation and data sharing between institutions and countries to ideally provide an open-source bone marrow database where independent ML models can be trained on analogous to existing cancer data bases such as The Cancer Genome Atlas [46]. Nevertheless, samples need to be properly anonymized to warrant patient data safety. If maintained and funded properly, such a database may vastly accelerate the development of clinically relevant computer vision tools for hematologic diagnostics. Standardization of data acquisition and accessible documentation of methodologies should be implemented to limit bias inherent to local methodologies of digitizing BMS and reporting patient data. Further, an integration of different diagnostic modalities such as cytomorphology both of bone marrow and peripheral blood, flow cytometry as well as genetic and clinical data seems warranted to build ML models that may aid in clinical decision making since evaluating only one modality at a time is insufficient for accurate diagnosis. Ensemble learning could be used to integrate the outputs of different ML models for different diagnostic modalities and provide a comprehensive and interpretable output to the clinician. Future work will focus on the extension of our ML pipeline for other use cases as well as different diagnostic modalities. As our study was limited to our center only, future studies will focus on transferability.

In conclusion, we here present a DL approach for the fast and accurate detection of AML from bone marrow cytomorphology. Our DL model accurately predicts *NPM1* mutation status and derived so far unreported morphologic features that indicate absence or presence of *NPM1* mutations from myeloblast morphology. This approach can be implemented to aid in clinical decision making, accelerate diagnosis, and may serve as a proof-of-concept for further studies of genetic imprints on disease morphology using DL.

## Data Availability

De-identified original BMS image data that supported the findings of this study are publicly available under https://www.kaggle.com/sebastianriechert/bone-marrow-slides-for-leukemia-prediction.

## Code availability

Python code for the DL models developed and implemented for the purpose of this study is publicly available under https://github.com/TimSchmittmann/DL_detection_of_AML_from_BMS and https://github.com/SebastianRiechert/autofrcnn and https://github.com/SebastianRiechert/npm1-training.
